# Human Papillomavirus prevalence and probable first effects of vaccination in 20 to 25 year-old women in Germany: a population-based cross-sectional study via home-based self-sampling

**DOI:** 10.1186/1471-2334-14-87

**Published:** 2014-02-19

**Authors:** Yvonne Deleré, Cornelius Remschmidt, Josefine Leuschner, Melanie Schuster, Michaela Fesenfeld, Achim Schneider, Ole Wichmann, Andreas M Kaufmann

**Affiliations:** 1 Immunisation Unit, Department for Infectious Disease Epidemiology, Robert Koch Institute, Seestraße 10 13353 Berlin, Germany; 2 Clinic for Gynaecology, Charité - Universitaetsmedizin Berlin, Berlin, Germany

**Keywords:** HPV, Cervical cancer, Genotype distribution, Self-sampling, Epidemiology

## Abstract

**Background:**

Estimates of Human Papillomavirus (HPV) prevalence in a population prior to and after HPV vaccine introduction are essential to evaluate the short-term impact of vaccination.

**Methods:**

Between 2010 and 2012 we conducted a population-based cross-sectional study in Germany to determine HPV prevalence, genotype distribution and risk factors for HPV-infection in women aged 20-25 years. Women were recruited by a two-step cluster sampling approach. A home-based self-collection of cervicovaginal lavages was used. Specimens were analysed using a general primer GP5+/GP6+-based polymerase chain reaction and genotyped for 18 high-risk and 6 low-risk HPV- strains by Luminex-based multiplexed genotyping.

**Results:**

Among 787 included women, 512 were not vaccinated against HPV. In the non-vaccinated population, HPV prevalence of any type was 38.1%, with HPV 16 (19.5%) being the most prevalent genotype. Prevalence of any high-risk type was 34.4%, and in 17.4% of all women, more than one genotype was identified. A higher number of lifetime sexual partners and low educational status were independently associated with HPV-infection. In 223 vaccinated women, prevalence of HPV 16/18 was significantly lower compared to non-vaccinated women (13.9% vs. 22.5%, p = 0.007). When stratifying by age groups, this difference was only significant in women aged 20-21 years, who at time of vaccination were on average younger and had less previous sexual contacts than women aged 22-25 years.

**Conclusion:**

We demonstrate a high prevalence of high-risk HPV genotypes in non-vaccinated women living in Germany that can be potentially prevented by vaccination. Probable first vaccination effects on the HPV prevalence were observed in women who were vaccinated at younger age. This finding reinforces the recommendation to vaccinate girls in early adolescence.

## Background

Persistent high-risk (hr) Human Papillomavirus (HPV) infections are essential for development of cervical precancer and cancer. Since 2006, two highly effective HPV vaccines have been available to prevent infections with hr-HPV genotypes 16 and 18. Many industrialised countries have adopted routine HPV vaccination into their national immunisation programmes [1]. Since 2007, all females in Germany aged 12-17 years have been eligible for vaccination free of charge [2]. Both, the quadrivalent and the bivalent vaccine are available in Germany.

Since it will take several decades until the effects of HPV vaccines on cervical cancer incidence will be visible, monitoring of HPV prevalence and genotype distribution prior to, and after vaccine introduction is essential to evaluate the short-term impact of HPV vaccination in a population. The prevalence of hr-HPV genotypes targeted by the vaccines is expected to decline after vaccine introduction. It remains unclear, however, to what extent the prevalence of certain other genotypes will change due to cross-protection or potentially by replacement by other genotypes.

### The objectives of this study were

(i) to establish a baseline HPV prevalence and genotype distribution in non-vaccinated women in Germany,

(ii) to identify factors associated with HPV infection in young women, and

(iii) to evaluate the feasibility of performing home-based sampling for HPV screening to investigate a population level genotype distribution.

In further analyses, we assessed if genotype distribution differs in a subgroup of vaccinated females compared to the non-vaccinated group.

## Methods

### Study design

We performed a cross-sectional study in Germany. Participants were recruited via invitation letters between October 2010 and September 2012.

### Study sample

The sampling methodology was based on the sampling protocol of the German Health Interview and Examination Survey for Adults (DEGS1) that has been described previously [3]. In brief, a two-step cluster sampling approach with communities as primary and individuals as secondary sampling units was used. First, communities were sampled from a list of German communities, stratified by districts, the grade of urbanisation, regional population density, and administrative borders according to the BIK classification system [4]. Sampling probability was proportionate to community size, and the Cox procedure for controlled rounding was used [5]. Within the primary sample points, individuals fulfilling the inclusion criteria were randomly drawn from local population registers.

Women who provided written informed consent for participation received a study package containing the self-sampling kit, an instruction leaflet, and a questionnaire.

### Representativeness of study sample

We compared our study sample of non-vaccinated women with the general population in Germany aged 20 to 25 years with respect to specific characteristics (i.e. migratory background, educational status, smoking and asthma prevalence) by using data (as of December 31^st^, 2010) provided by the German Federal Statistical Office (Destatis) [6] or results of a representative survey (“GEDA”) of the German population in 2010 [7].

### Self-sampling and HPV testing

Self-sampling was performed by cervicovaginal lavage (5 ml volume) with the first generation Delphi-Screener (Delphi-Bioscience, Scherpenzeel, The Netherlands) as described previously [8]. General primer GP5+/GP6+-based polymerase chain reaction (PCR) was applied and genotyping was performed using Luminex-based multiplexed genotyping (MPG, multiplexed HPV genotyping, Multimetrix, Heidelberg, Germany) for detection of 18 hr-HPV genotypes (HPV 16, 18, 26, 31, 33, 35, 39, 45, 51, 52, 53, 56, 58, 59, 66, 68, 73 and 82 and 6 lr-HPV genotypes (HPV 6, 11, 42, 43, 44, and 70) [9]. Women were asked not to participate in the study if they were pregnant at time of the study (for safety reasons).

### Questionnaire and definitions

A 23-item questionnaire was used to collect the following data:

•Demographic data (e.g. age, migratory background (i.e. at least one parent who immigrated into the country), education);

•Data on sexual behaviour (e.g. age in years at first intercourse, number of lifetime sexual partners and during the last 12 months, condom use during one-night stands, living in a relationship);

•Medical history (e.g. previous diagnosis of genital warts, history of chronic diseases, participation in cervical cancer screening, pregnancies);

•Smoking habits (e.g. active or past smoker, number of cigarettes per day, years of smoking);

•Data on HPV-vaccination (e.g. number of doses and dates of vaccination (month/year), vaccine brand);

•Acceptability of the self-sampling method, i.e. score from 1 (very easy/convenient) to 6 (very difficult).

According to the hierarchical structure of the German school system, educational status was divided into 3 categories: Low educational status was defined as no school-leaving degree, or a qualification acquired after 5 years at a secondary school. Medium educational status was defined as graduation after 6 years at secondary school, and high educational status was defined as 8 or 9 years at secondary school leading to a general qualification for university entrance called “Abitur”, or a university degree. To assess the acceptance of the self-sampling method we applied a score from 1 (very easy/convenient) to 6 (very difficult). Data from the questionnaires were self-reported and not validated. We defined 'young age at first intercourse’ as sexual debut before 14 years of age, which corresponds to the 20th percentile of the distribution of this variable. Vaccination was defined as receipt of ≥ 1 dose. The variable “mean time delay [years] between first intercourse and first dose of vaccination” was calculated by subtracting age at vaccination (first dose) from age at first intercourse among all vaccinated women with evaluable information (n = 173).

### Sample size calculation

We expected an all-type HPV prevalence of 45% based on data from our pilot study conducted in 2010 and from a large Danish population-based study [8,10]. We calculated a required sample size of 780 women in order to obtain a high precision of the estimate (± 0.05) regarding all-type HPV prevalence. With an expected response of 15%, in a first step we invited 5,200 women to participate in our study. If the required sample size would not be achieved, in a second step up to 1,500 more invitations were planned.

### Statistical analysis

We performed descriptive analyses of HPV prevalence and genotype distribution both in non-vaccinated and vaccinated women, and reported results as percentages with 95% confidence intervals (CI). Risk factors for HPV infection of any or of specific HPV types or groups (i.e. hr or lr) were assessed. We performed chi-square tests and Fisher’s exact test for categorical, and Student’s *t*-test for numerical variables. We conducted univariate analyses for all binary or categorical exposure variables and calculated crude odds ratios (OR) and 95% CI. Variables with a p-value <0.2 in univariate analysis were included in an initial step for multivariate modelling, followed by stepwise backward removal of all variables with a p-value >0.05 to obtain the final model. The statistical software package STATA®, version 11 (STATA Corp., College Station, TX, USA) was used.

### Ethical considerations

The study was approved by the local ethics committee (Charité, Universitätsmedizin-Berlin, EA2/028/10) and registered at German Clinical Trials Register (DRKS00000599).

## Results

**Figure 1:**
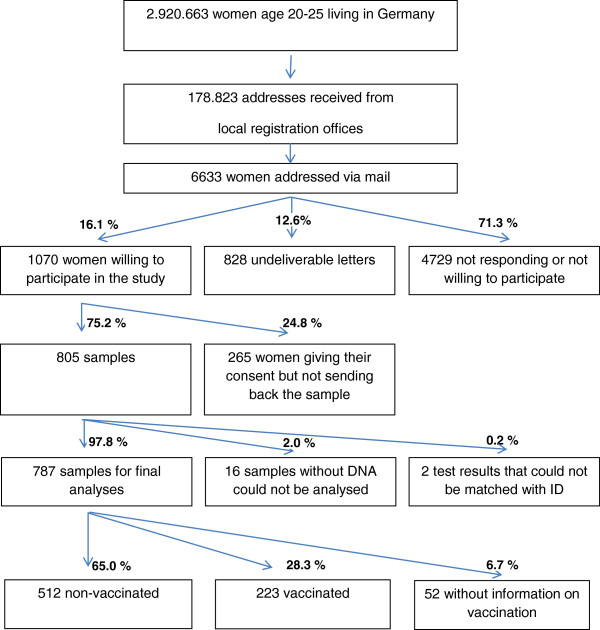
Flow chart for the recruitment of the study population.

In total, 787 (11.9%) women agreed to participate in the study and returned an adequate cervicovaginal lavage specimen. Of those, 223 (28.3%) reported to be vaccinated against HPV, 512 (65.0%) were not vaccinated and 52 (6.7) missed to report their HPV-vaccination status (Figure 1).

Regarding the vaccinated group, 198 of 223 (88.8%) participants reported of having received three doses, 14 (6.3%) two doses and 11 (4.9%) one dose, respectively. The quadrivalent vaccine was administered in 194 of 223 individuals (87%).

### HPV prevalence and genotype distribution in the non-vaccinated population

Key characteristics of the non-vaccinated (n = 512) and the vaccinated (n = 223) study population and the general female population of the same age group are presented in Table 1. The educational status was the only characteristic of the non-vaccinated study sample that considerably differed from that of the general population. A low educational status was reported by 6.1% of participants compared to 22.2% of the general female population and a high educational status by 64.4% and 44.9%, respectively. Age of participants ranged from 20 to 25 years (mean, 23.0 years, standard deviation ± 1.4). Two hundred and fourteen (48.2%) had participated at least once in the cervical cancer screening programme.

**Table 1:** Characteristics of 512 non-vaccinated and 223 vaccinated women and the general female population in Germany

	**Study sample (non-vaccinated women, n = 512)**	**Study sample (vaccinated women, n = 223)**	**General female population age 20-24 years, Germany**
	**% (95% CI)**	**% (95% CI)**	**%**
Residency^*1^			
Western federal states	81.7 (78.1-85.0)	87.8 (82.7-91.8)	83.7 ^*2^
Eastern federal states	18.3 (15.0-21.9)	12.2 (8.2-17.3)	16.3 ^*2^
**Size of residency**			
< 500,000 inhabitants	82.3 (78.7-85.5)	84.6 (79.2-89.1)	80.5
≥ 500,000 inhabitants	17.7 (14.5-21.3)	15.4 (10.9-20.8)	19.5
Migratory background^*3^			
No	81.7 (78.1-85.0)	94.5 (90.6-97.1)	78
Yes	18.3 (15.0-21.9)	5.5 (2.9-9.4)	22
Educational status^*1^			
Low	6.1 (4.2-8.5)	1.4 (0.3-3.9)	22.2
Medium	29.5 (25.5-33.6)	26 (20.4-32.3)	32.8
High	64.4 (60.1-68.6)	72.7 (66.3-78.4)	44.9
Active smoker^*3^			
No	65.5 (61.2-69.6)	83.4 (77.9-88.0)	67.2
Yes	34.5 (30.4-38.8)	16.6 (12.0-22.1)	32.8
**History of asthma**			
No	93.5 (91.0-95.5)	95.5 (92.0-97.8)	91.1^*4^
Yes	6.6 (4.6-9.2)	4.5 (2.2-8.1)	8.9 ^*4^

95% CI: 95% confidence interval.

*1 p-value <0.05; *2 comparison group: women age 20-25 years; *3 p-value <0.001; *4 women aged 18-29 years.

**Figure 2:**
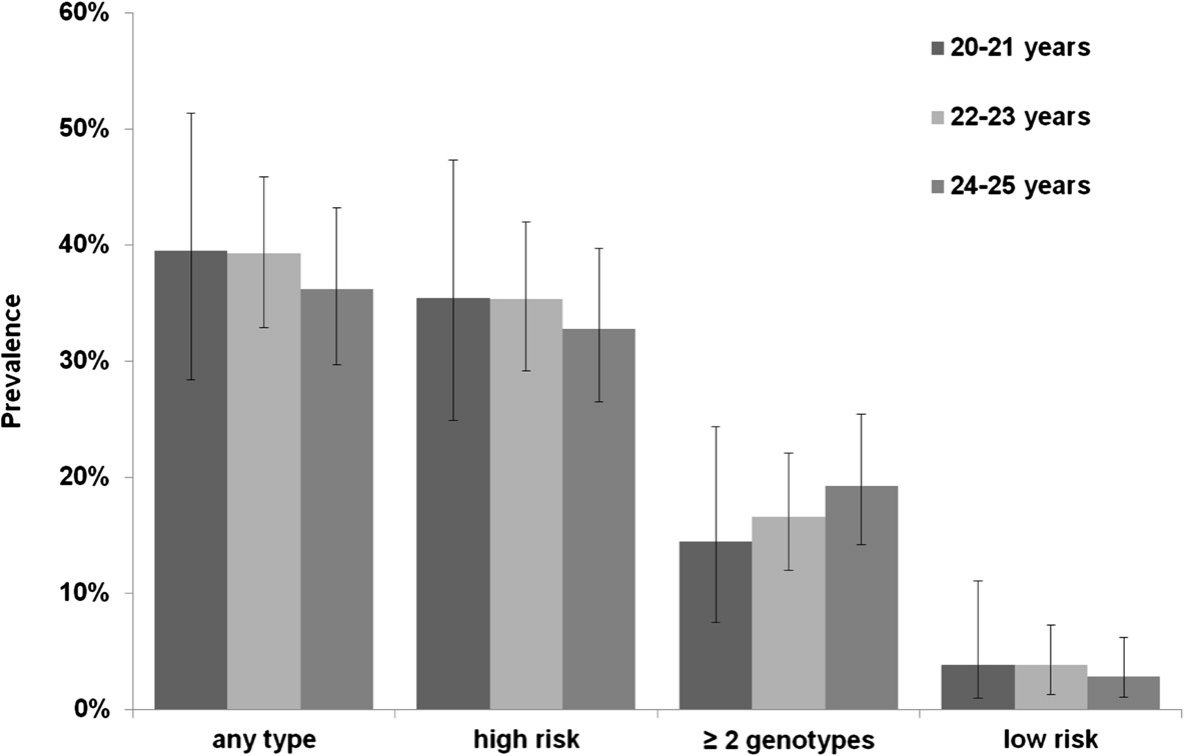
HPV prevalence of any type, high- and low-risk types and multiple infections among HPV non-vaccinated women (n = 512) by age group.

The most commonly detected HPV genotypes in the non-vaccinated population are presented in Table 2. The prevalence of HPV infection of any type was 38.1% (95% CI: 33.9-42.4), 34.4% (95% CI: 30.3-38.7) were infected with at least one high-risk genotype, 22.5% (95%CI, 18.9-26.3) with HPV type 16 or 18, 3.5% (95% CI:2.1-5.5 ) with low-risk genotypes only, and in 17.4% (95% CI: 14.2-20.9) multiple infections with more than one genotype were identified. One hundred and five (20.5%, 95% CI: 17.1-24.3) women were infected with one genotype only, 50 (9.8%, 95% CI: 7.3-12.7) with two, 28 (5.5%, 95% CI: 3.7-7.8) with three and 11 (2.2%, 95% CI: 1.1-3.8) with more than three HPV genotypes. Out of 89 women infected with more than one genotype, all but one (98.9%, 95% CI: 94.0-100) were infected with at least one hr-type. Differences in the prevalence of any HPV type between the three age groups 20-21, 22-23 and 24-25 years were statistically not significantly different. They ranged from 39.9% (95% CI: 28.4-51.4) in women aged 20 to 21 years to 36.2% (95%CI, 29.7-43.2) in women aged 24 to 25 years (Figure 2). Likewise, there were no statistically significant differences in the prevalence of hr-types or the detection of multiple infections between the three age groups. Hence, we report pooled prevalences for all age groups.

**Table 2:** Prevalence of specific HPV types and groups of types in 512 HPV non-vaccinated women, Germany 2010-2012

**HPV type**	**No (%) of 512 non-vaccinated women tested**	**95% CI**
Any type	195 (38.1)	33.9; 42.4
High-risk type	176 (34.4)	30.3; 38.7
≥ 2 genotypes	89 (17.4)	14.2; 20.9
**16**	**100 (19.5)**	**16.2; 23.2**
**53**	**39 (7.6)**	**5.4; 10.3**
42	31 (6.1)	4.1; 8.5
**18**	**26 (5.1)**	**3.3; 7.3**
**56**	**19 (3.7)**	**2.2; 5.7**
**66**	**19 (3.7)**	**2.2; 5.7**
**51**	**16 (3.1)**	**1.8; 5.0**
**39**	**15 (2.9)**	**1.6; 4.8**
**59**	**15 (2.9)**	**1.6; 4.8**
**52**	**11( 2.2)**	**1.1; 3.8**
6	5 (1.0)	0.3; 2.3
**70**	**5 (1.0)**	**0.3; 2.3**
**58**	**5 (1.0)**	**0.3; 2.3**
82	5 (1.0)	0.3; 2.3
11	4 (0.8)	0.1; 2.0
43	4 (0.8)	0.1; 2.0
**33**	3 (0.6)	0.1; 1.7
44	3 (0.6)	0.1; 1.7
**73**	**3 (0.6)**	**0.1; 1.7**
**31**	**2 (0.4)**	**0.1; 1.4**
**68**	**2 (0.4)**	**0.1; 1.4**
**26**	**1 (0.2)**	**0.0; 1.0**
**35**	**1 (0.2)**	**0.0; 1.0**
**45**	**1 (0.2)**	**0.0; 1.0**

95% CI: 95% confidence interval.

### Factors associated with HPV infection in the non-vaccinated population

In univariate analysis, a higher number of lifetime sexual partners significantly increased the probability of HPV infection by any type and by hr-types. Furthermore, women with 2 or more sexual partners during the past 12 months had a significantly increased risk of HPV infection compared to those who had no partners during this time interval. Also current smokers had an increased risk of being HPV positive. In contrast, preference for condom use during one-night stands and a higher education significantly reduced the chance of being HPV positive (Table 3). Four out of 20 women (20%), who reported not yet having had vaginal intercourse, were still found to be HPV positive.

**Table 3:** HPV prevalence (any type) and risk factors for HPV detection (univariate analysis) in 512 non-vaccinated women, Germany 2010-2012

**Variable and category**	**No (%) of women with characteristic**	**No (%) of women with HPV infection by presence of characteristic**	**Crude OR (95% CI)**	**p**
**Age group in years**	512 (100)	195 (38.1)		
20-21	76 (14.8)	30 (39.5)	1	-
22-23	229 (44.7)	90 (39.3)	1.0 (0.6-1.7)	1
24-25	207 (40.4)	75 (36.2)	0.9 (0.5-1.5)	0.6
**Migratory background**	509 (99.4)			
No	416 (81.7)	158 (38.0)	1	-
Yes	93 (18.3)	35 (37.6)	1.0 (0.6-1.6)	0.95
**Educational status**	509 (99.4)			
Low	31 (6.1)	19 (61.3)	1	-
Medium	150 (29.5)	55 (36.7)	0.4 (0.2-0.8)	0.01
High (i.e. high school diploma)	328 (64.4)	119 (36.3)	0.4 (0.2-0.8)	0.001
**Current smoker**	510 (99.6)			
No	334 (65.5)	116 (34.7)	1	-
Yes	176 (34.5)	77 (43.8)	1.5 (1.0-2.1)	0.05
**Participation in cervical cancer screening**	444 (86.7)			
No	230 (51.8)	78 (33.9)	1	-
Yes	214 (48.2)	87 (40.7)	1.3 (0.9-2.0)	0.14
**Living in a relationship**	512 (100)			
No	131 (25.6)	52 (39.7)	1	-
Yes	381 (74.4)	143 (37.5)	0.9 (0.6-1.4)	0.74
Age at first intercourse^*1^	488 (95.3)			
≤ 14	97 (19.9)	40 (41.2)	1	-
> 14	391 (80.1)	149 (38.1)	0.8 (0.5-1.3)	0.46
**Lifetime sexual partners**	508 (99.2)			
0	20 (3.9)	4 (20.0)	1	-
1-2	145 (28.5)	28 (19.3)	1.0 (0.3-3.0)	0.94
3-5	168 (33.1)	63 (37.5)	2.4 (0.8-7.5)	0.1
6-10	111 (21.9)	54 (48.7)	3.8 (1.2-12.0)	0.02
>10	64 (12.6)	43 (67.2)	8.2 (2.4-27.5)	0.001
**Sexual partners in the past 12 months**	509 (99.4)			
0	41 (8.1)	10 (24.4)	1	-
1	353 (69.4)	107 (30.3)	1.3 (0.6-2.8)	0.4
≥ 2	115 (22.6)	76 (66.1)	6.0 (2.7-13.6)	<0.001
**History of genital warts**	499 (97.5)			
No	485 (97.2)	183 (37.7)	1	-
Yes	14 (2.8)	8 (57.1)	2.2 (0.8-6.4)	0.1
**Condom use during ONS**	508 (99.2)			
Not always	98 (19.3)	53 (54.1)	1	-
Yes, always	410 (80.7)	140 (34.2)	0.4 (0.3-0.7)	<0.001
Current use of oral contraceptives*^2^	334 (65.2)			
No	82 (24.5)	27 (32.9)	1	-
	252 (75.5)	90 (35.7)	1.1 (0.7-1.9)	0.6

OR, odds ratio; 95% CI, 95% confidence interval; ONS, one-night stand.

*1 only women with sexual intercourse.

*2 only women with current use; overall, 452 (88.2%) of 512 women reported use of oral contraceptives at any time.

The final multivariate model indicated that an increasing number of lifetime sexual partners and lower educational status were independently associated with the presence of HPV infection. Adjusting for age did not substantially affect the model.

### Acceptance of the self-sampling method in the non-vaccinated population

Three hundred seventy-six women (73.4% (95% CI: 69.4-77.2)) rated the home-based self-sampling method as “very easy” or “easy” (“very easy”: 35.2%; “easy”: 38.3%) and less than 1% (95% CI, 0.2-2.0) as “difficult” or “very difficult”). Two hundred forty-one women (47.1%, 95% CI: 42.7-51.5) stated that they would prefer self-sampling over sampling by a clinician in the future, whereas 187 women (36.5% 95% CI: 32.3-40.9) would prefer sampling by a physician. 14.3% of the remaining participants did not report a preferred method (“do not know”) and 2.2% did not answer. Women who preferred physician sampling were more likely to have prior participated in the cervical cancer screening programme than those who preferred self-sampling at home (p < 0.01).

### Comparison of HPV prevalence in vaccinated and non-vaccinated women

The prevalence of HPV 16/18 in women who reported to be vaccinated (≥1 doses) was significantly lower than in non-vaccinated women (13.9% vs. 22.5%, p = 0.007). When stratifying by age groups, this difference was only significant in the age group 20-21 years (p = 0.016), but not in the other age groups (Table 4). Average age at receipt of the first HPV vaccine dose was 16.7 years for the age group 20-21 years and increased up to an average of 20.8 years in the oldest age group (24-25 years). Among 177 of 223 (79.4%) vaccinated women with information on both, age at first sexual intercourse and age at time of vaccination, 54 reported never having had sex before vaccination. The proportion of women without sexual debut at time of vaccination decreased by age-group: 42.2%, 20% and 4% in age groups 20-21, 22-23 and 24-25 years, respectively (p < 0.001). Moreover, the mean time delay between first intercourse and the first dose of vaccination was 0.5 years in 20-21-year-olds, 1.6 years in 22-23-year-olds, and 4.3 in 24-25-year-old women, respectively. Among the 54 women who reported having had 1^st^ HPV vaccination prior to sexual debut only 5 (9.3%) were positive for either HPV-type 16 or 18.

**Table 4:** Prevalence of HPV 16/18 in both vaccinated (n = 223) and non-vaccinated women (n = 512) by age group, Germany 2010-2012

**Age group**	**n**	**n vaccinated (%)**	**HPV 16/18 prevalence ** **in non-vaccinated women % (95% CI)**	**HPV 16/18 prevalence in vaccinated women % (95% CI)**	**p**	**Mean age [years] at vaccination among vaccinated (n = 188)**	**Mean time delay [years] between first intercourse and first dose of vaccination (n = 173)**
**All age groups**	735	223 (30.3)	22.5 (18.9-26.3)	13.9 (9.6-13.1)	0.007	17.7	1.3
**20-21**	213	137 (64.3)	22.4 (13.6-33.4)	10.2 (5.7-16.6)	0.016	16.7	0.5
**22-23**	288	59 (20.5)	22.7 (17.4-28.7)	20.3 (11.0-32.8)	0.70	18.2	1.6
**24-25**	234	27 (11.5)	22.2 (16.7-28.5)	18.5 (6.3-38.1)	0.66	20.8	4.3

95% CI, 95% confidence interval.

Some key characteristics, which are potentially associated with an increased risk for HPV detection, were more frequently found in the population of the non-vaccinated compared to vaccinated women, namely a higher mean number of sexual partners (5.8 vs. 3.8, p < 0.001), smoking (34.5% vs. 16.6%, p < 0.001) and an immigrant background (18.3% vs. 5.5%, (Table 1)). After adjustment for these risk factors in a multivariate model, HPV vaccination was still significantly associated with a lower risk for HPV 16/18 infection in the youngest age group (OR = 0.44, p = 0.045). However, for the older age groups there was no significant association between vaccination status and a lower HPV 16/18 prevalence (data not shown).

## Discussion

We used a cervicovaginal lavage self-sampled at home to determine population-based HPV prevalence in young women in Germany. In non-vaccinated women aged 20-25 years, HPV prevalence of any type was almost 40% and prevalence of hr-types was 34%. Among participants aged 20-21 years, HPV 16/18 prevalence was significantly lower in women who reported to be vaccinated than in non-vaccinated women. Women in this group also had lower prevalence of risk factors, but after adjustment for these risk-factors, HPV vaccination was still significantly associated with a lower risk for HPV 16/18 infection in the youngest age group.

Additionally, the quadrivalent vaccine was administered in 87% of participants. Sales data for HPV vaccines in Germany confirm a preference for the quadrivalent vaccine of >90% in 2009 [11].

Large population-based studies in Denmark and the US found HPV prevalences of 50.2% and 53.8% in non-vaccinated women aged 20-24 years, respectively [10,12]. In contrast, a prevalence of 28.3% of any HPV type was identified in a sample of 354 women aged 20-22 years in a previous German study [13]. The authors of another recently published German study (WOLVES) demonstrated an hr-HPV prevalence of 23.7% in 599 women born 1988/1989 with a first residency in the city of Wolfsburg [14]. The observed prevalence differences might result from different study populations, sampling methods, and tests utilized for HPV detection. Iftner et al. recruited participants at office-based gynecologists when attending for routine cytological screening [13]. Since only about half of 20-29-year-old women participate in annual cervical cancer screening in Germany [15], this selection might have biased results. Furthermore, three studies used a hybrid capture (HC2) test for prescreening and a line probe assay that detects 24 HPV types [10,13,14]. The study conducted in the US used a linear array HPV genotyping test that detects a wider range of 37 HPV types [12]. Generally, the analytic sensitivity of the HC2 assay seems to be relatively low [16]. The purpose of the HC2 was to detect clinically important infections. For the usage in epidemiological studies, PCR amplification - such as the multimetrix test used in our study – could significantly increase sensitivity of HPV detection assays [17].

Consistent with other data, a higher number of lifetime sexual partners was an independent risk factor for HPV infection [14,18]. In addition, a lower educational status was associated with a higher HPV prevalence. Low educated women were underrepresented in our study, but this finding is supported by a large study from the US, where a lower educational status was a predictor of HPV detection [18]. In our study other factors that have been described previously to be associated with HPV infection, such as active smoking, young age at first intercourse, or a higher number of sexual partners in the past 12 month, were only significant risk-factors in univariate analysis [13,18]. It is possible that our study had limited power to detect these risk-factors or these factors are confounders. It has been shown that active smoking or young age at sexual debut are associated with a higher number of sexual partners [19,20]. It is important to note, however, that factors associated with the presence of HPV infection may not be identical with factors associated with a risk of developing cervical abnormalities [21]. Genotype of infection, viral load, and individual health conditions might contribute to a higher risk of cervical precancer and cancer as well [22-27].

Among our participants aged 20-21 years, HPV 16/18 prevalence was significantly lower in vaccinated than in non-vaccinated women. These findings are in line with recently published studies from Australia and the US [28,29]. The lack of significant differences in HPV prevalence in the higher age groups in our study is likely due to the fact that most vaccinated women in these age groups were vaccinated long after first sexual intercourse. This might have negatively impacted on vaccine effectiveness [30]. These findings support the recommendation to vaccinate early in adolescence and before sexual debut.

Some limitations of our study need to be mentioned. First, the response to participate in our study was only about 12%, potentially introducing selection bias. Women who are interested in health-related issues or feel they may be at risk could be higher attracted to participate in a study like this. Second, information on sexual behaviour, vaccination status and other characteristics were self-reported and could be subject to misclassification. However, agreement statistics of self-reported vaccination status compared to electronic medical records (EMR) have been more favourable for HPV than for other vaccines (e.g. Tetanus, Hepatitis B) [31]. Nevertheless, neither sensitivity nor specificity of self-reported vaccination status versus EMR was 100%. Third, since more participants than expected had already been vaccinated against HPV, we failed in obtaining the calculated sample size. Additionally, the sample size was calculated to establish the overall HPV prevalence. This might have hampered power of statistical analyses. Fourth, in our study population many females received the HPV vaccination several years after their sexual debut. Hence, the real impact of the vaccine is likely to be underestimated. Finally, the cervicovaginal lavages included the whole vaginal area, wherefore additional infections at extracervical sites might have influenced our HPV prevalence estimates.

## Conclusion

We found a high HPV prevalence in young non-vaccinated women aged 20-25 years in Germany, with the number of lifetime sexual partners and low educational status being risk factors for present infection. A significantly lower prevalence of HPV 16/18 was observed in vaccinated women suggesting first effects of vaccination. The finding that HPV 16/18 prevalence was lowest in the youngest women, 40% of whom had received their first vaccination dose before starting sexual activities, reinforce the recommendation to vaccinate girls in early adolescence and before sexual debut.

These data provide a useful baseline HPV prevalence estimate shortly after the introduction of prophylactic HPV vaccination in Germany and are essential to evaluate the impact of HPV vaccination on HPV prevalence and type distribution in forthcoming years. For this purpose, prevalence studies should be repeated in the near future with the same study design and HPV-tests to allow for a direct comparison with these baseline data. Self-sampling at home via cervicovaginal lavage provides a method for monitoring that is highly accepted by study participants and allows easy inclusion of women from all parts of the country even if not attending the screening programme.

## Abbreviations

CI: Confidence interval; DEGS: German health interview and examination survey for adults; DRKS: German clinical trials register; GEDA: German health update survey; HPV: Human papillomavirus; hr: high risk; lr: low risk; OR: Odds ratio; RKI: Robert Koch Institute.

## Competing interests

The study was financed by internal funds of the Robert Koch Institute. Andreas Kaufmann (AMK) has contributed as expert and member to advisory committees for GlaxoSmithKline (GSK) and Gen-probe. He has received speaker’s honoraria and travel support to conferences from Sanofi Pasteur MSD, GSK and Roche. The remaining authors declare no competing interests.

## Authors’ contributions

YD was responsible for the overall study design, study performance, data collection and interpretation and manuscript writing. CR performed data collection, statistical analysis, interpretation and manuscript writing. JL carried out HPV testing. MS was responsible for recruitment and trial coordination. MF performed statistical analysis, AS and OW gave important intellectual content and revised the manuscript critically. AMK carried out HPV testing and data collection. All authors read and approved the final manuscript.

## Pre-publication history

The pre-publication history for this paper can be accessed here:

http://www.biomedcentral.com/1471-2334/14/87/prepub
